# Lipoprotein(a): Assessing the Current Knowledge and Gaps in Screening and Treatment—A Narrative Review

**DOI:** 10.3390/jcdd12050169

**Published:** 2025-04-26

**Authors:** Octavian Amaritei, Oana Laura Mierlan, Cristian Gutu, Gabriela Gurau

**Affiliations:** 1 Faculty of Medicine and Pharmacy, “Dunărea de Jos” University of Galați, 800008 Galați, Romania; laura.mierlan@ugal.ro (O.L.M.); gabriela.gurau@ugal.ro (G.G.); 2 “Sf. Andrei” Clinical Emergency County Hospital, 800578 Galați, Romania; 3 “Sf. Ioan” Emergency Clinical Pediatric Hospital, 800487 Galați, Romania; 4 “Dr. Aristide Serfioti” Emergency Military Hospital, 800150 Galați, Romania; 5 Center for Research and Technology Transfer in the Medico-Pharmaceutical Field, "Dunărea de Jos" University of Galați, 800008 Galați, Romania

**Keywords:** lipoprotein(a), LDL cholesterol, cardiovascular disease, atherosclerosis, PCSK9-i, ASO, siRNA

## Abstract

Atherosclerotic cardiovascular disease (ASCVD) has long been screened using the traditional lipid profile, mainly focusing on LDL cholesterol. However, despite growing evidence supporting lipoprotein(a) [Lp(a)] as an independent risk factor involved in atherosclerosis, its clinical use remains limited. This review examines the reasons behind the limited use of Lp(a) screening in clinical practice, assessing its role in cardiovascular risk, comparing it to traditional lipid markers and evaluating current assessment methods. It also explores existing and emerging treatments, including gene-silencing therapies, for managing elevated Lp(a) levels. One in four clinicians does not routinely check Lp(a) levels, which proves a lack of awareness amongst them. The reasons for that are implied to be that the cost is too high and that available treatments are scarce. The traditional lipid profile, including LDL, high-density lipoprotein (HDL) and triglycerides, continues to be the gold standard for CV risk assessment. One limitation of using Lp(a) in clinical practice is the significant variability in apo(a) sizes, which results from the presence of multiple isoforms determined by the number of kringle domains. This structural diversity poses challenges in standardizing measurement methods, affecting the accuracy and comparability of results. While statins have a minimal impact on Lp(a), PCSK9-i lowers its levels by 20–25%, although this class is not prescribed primarily for this reason. Lastly, gene-silencing therapies, which achieve the greatest reduction in Lp(a) levels, are still in phase III trials, and there is still a need to examine whether this reduction translates into CV benefits. These limitations should not discourage further research, because ASCVD’s complexity requires a more tailored approach. Current lipid-lowering therapy still fails in a minority of cases, as evidenced by new-onset cardiovascular events in patients with well-controlled LDL levels. There is a need for future interventional studies to assess whether a reduction in Lp(a) by PCSK9-i really translates into CV benefits, independent of LDL.

## 1. Introduction

In recent years, cardiovascular disease (CVD) has remained the leading cause of mortality worldwide, with atherosclerosis being one of the culprits [1]. Traditional risk factors, such as diabetes, dyslipidemia and hypertension, have been proven to contribute to the development of atherosclerotic plaque. However, the focus has started shifting towards new, promising risk factors, including adhesion molecules, pro-inflammatory molecules, oxidized phospholipids and Lp(a) [2,3].

The traditional lipid profile has been used for decades as an important tool for screening for ASCVD. However, recent studies have linked atherosclerosis to inflammation and immune processes, suggesting that LDL is no longer the sole driver for the development of atherosclerosis. Furthermore, the deposition of cholesterol particles is now thought to be an active phenomenon, rather than a passive one [3].

There are many limitations to the use of Lp(a) as a potential biomarker. Amongst them, the lack of current validated treatments is a major one. Studies evaluating the efficacy and safety of antisense oligonucleotides (ASOs) are still ongoing, with results expected to be available in the coming years. However, even if we surpass this limitation, we still have a subtle but significant challenge: the myriad of apo(a) isoforms, which directly influence the structure of Lp(a) and make current screening tests less reliable due to a lack of standardization [4]. This highlights the importance of carefully evaluating whether Lp(a) has been measured using the most reliable methods in trials where lowering Lp(a) was the primary objective.

The genetic inheritance of an Lp(a) level and the absence of its fluctuation with lifestyle or current treatments have diminished efforts toward its future research, and instead, attention has shifted back to LDL, exploiting it even more. The initial reduction in the mean LDL determined by alirocumab in the Odyssey Outcomes trial waned after 4 months and even further by the 48-month mark, but its positive CV outcomes persisted, suggesting that other mechanisms influenced by PCSK9-i underlie these outcomes [5,6].

The purpose of this review is to emphasize the urgent need for a standardized method in determining Lp(a), given the variety of apo(a) isoforms, which in turn discourages screening in clinical practice [7,8]. It also highlights the insufficient research into the relationship between PCSK9-i and Lp(a) [5]. By improving our knowledge and guiding research in this direction, Lp(a) could move closer to a clinical implementation alongside the traditional lipid profile.

## 2. Materials and Methods

### 2.1. Research Strategy

We conducted a search through international databases such as PubMed, Google Scholar, ResearchGate, Scopus and Web of Science, with an emphasis on randomized clinical trials, cohort studies, case–control studies, systematic reviews and meta-analyses published in English in the last 5 years. Keywords such as ‘lipoprotein(a)’, ‘CV risk’, ‘PCSK9-i’, ‘antisense oligonucleotides’ and ‘atherosclerosis’ were used to refine the search process and to ensure adequate results. Ongoing clinical trials were also reviewed, highlighting the methodologies used for Lp(a) serum determination and the study outcomes. Last but not least, based on the results, we outlined future directions that could serve as a starting point for translating theoretical findings to practical applications. This review follows PRISMA (Preferred Reporting Items for Systematic Reviews and Meta-Analyses) guidelines.

### 2.2. Inclusion and Exclusion Criteria

The selection of studies was based on predefined inclusion and exclusion criteria. Only studies focusing on lipoprotein(a) measurement, screening methods, its role in cardiovascular disease and potential therapies were included.

Exclusion criteria included non-peer-reviewed sources, animal studies, duplicate publications and those with incomplete data. Studies older than the specified period and that did not provide additional information were also excluded.

### 2.3. Study Selection

The initial search yielded 2196 results. Following a triage based on titles and abstracts, 56 studies were selected. Despite these containing the most recent data, some relevant information was still missing. To address this, the timeframe was extended by five years (2014–2024), resulting in the inclusion of 15 additional studies. However, there were consistent gaps regarding certain screening methods. To comprehensively understand these limitations, six more studies were added, with the oldest dating back to 2000.

## 3. Lipoprotein(a): Structure and Pathophysiology

Lipoprotein(a) stands out among lipoproteins due to its unique structure, consisting of apo-B100, an LDL-like particle, which covalently binds to apo(a), a plasminogen-like particle, through a disulfide bond [4,9]. Therefore, Lp(a) combines the pro-inflammatory and atherogenic properties of LDL with an additional prothrombotic effect, primarily due to the structural resemblance between apolipoprotein(a) and plasminogen, which disrupts normal fibrinolysis [10,11,12,13]. Its physiological role is yet to be discovered, but since it resembles plasminogen, it might act as an antifibrinolytic factor [4,14]. Apo(a) is composed of kringle domains (KIV and KV), which vary in size and exist in numerous isoforms. This variability in apo(a) structure ultimately leads to the size differences observed in Lp(a) [4,9].

Genetic traits are the most recognized factors that play a role in the variability in Lp(a), accounting for almost 90% of it [15]. This is also the reason why Lp(a) levels remain fairly constant throughout an individual’s life but vary significantly between individuals. Individuals of African descent have higher serum concentrations compared to Caucasians [16]. Kamal Awad et al. [17] published a study in 2024, stating that there is a relationship between Lp(a) and other CV risk factors. The highest individual variability in Lp(a), defined as >10 mg/dL, was observed in individuals with high LDL, those undergoing statin treatment and those with a history of CVD. Lp(a) concentration is mostly regulated on the production site. To make the most impact, this factor must be considered in the development of future treatments. The primary target for medication should be the production site, specifically the liver [14,17,18].

One of the key features of Lp(a) is increasing the expression of pro-inflammatory cytokines, such as Interleukin-6 (IL-6), Interleukin-8 (IL-8) and Tumor necrosis factor-α (TNF-α) and adhesion molecules, like E-selectin, Vascular cell adhesion molecule-1 (VCAM-1) and P-selectin. This promotes the chemoattraction of immune cells, thereby amplifying inflammation and ultimately contributing to the progression of atherosclerosis [19,20,21]. Lp(a) also carries approximately 70% of plasma-oxidized phospholipids, which directly influence plaque composition, making it more unstable and, consequently, more prone to rupture. Patients with elevated Lp(a) levels are more likely to present with acute rather than chronic coronary syndrome [3,21,22,23].

## 4. Cardiovascular Risk

A Mendelian randomization study published in 2016 by Emdin C.A. et al. [24] found that by genetically lowering Lp(a), the risk for heart disease is reduced. There was a risk reduction by 37% for aortic stenosis, 31% for peripheral artery disease, 29% for coronary heart disease, 17% for heart failure and 13% for stroke. The study included data from 112,338 participants and had some significant limitations, including the reliance on patients’ reports regarding their past medical history rather than verifying medical records and the relatively low variability regarding ancestry—the majority of the subjects were of European descent [24].

Data from two Danish studies, which included a total of 98,097 subjects, were analyzed by Pia R. Kamstrup et al. [25], and the results suggested that the opposite is also true—high Lp(a) levels increase the risk of heart failure by 18%, mostly because of the effect of Lp(a) on other cardiovascular diseases like myocardial infarction and valvular disease. The diagnosis of heart failure was obtained from the patient’s history, without conducting new echocardiographic studies to evaluate ventricular function at the time of the enrollment [25].

A study of 2527 subjects with established CVD, published in 2019 by Christian M. Madsen et al. [26], evaluated the risk of major cardiovascular events (MACEs) during a median follow-up of 5 years. MACE rates ranged from 29 per 1000 person-years for an Lp(a) < 10 mg/dL to 54 per 1000 person-years for an Lp(a) ≥ 100 mg/dL. Also, the study showed that in secondary prevention, a 20% or 40% reduction in MACE could be achieved by an Lp(a) reduction of 50 mg/dL and 99 mg/dL, respectively. More than one type of assay was used in this study to determine serum Lp(a) levels, and although results were calibrated, this still predisposes it to inconsistent measurements [26,27].

A meta-analysis of 29,069 patients, including 8064 women, with repeat Lp(a) measurements, found a linear association between both baseline and on-statin Lp(a) levels and cardiovascular disease risk, with an increased risk observed at Lp(a) levels of 30 mg/dL or higher for baseline and 50 mg/dL or higher for on-statin Lp(a) [28].

## 5. Prevention

Recent studies have demonstrated that while both elevated Lp(a) levels and coronary artery calcium (CAC) scores are associated with increased cardiovascular risk, there appears to be no direct correlation between the two. Lp(a) is predominantly linked to non-calcified, high-risk plaques that do not contribute to the CAC score. Therefore, in patients presenting with angina, risk stratification should not rely solely on CAC scoring, as it may overlook a significant burden of unstable, rupture-prone plaques characterized by thin fibrous caps, which are potentially more dangerous [29,30].

Although antiplatelet therapy for the primary prevention of major adverse cardiovascular events (MACEs) lacks sufficient evidence and may even pose risks, patients with an elevated Lp(a) and low bleeding risk could represent an exception. These individuals might benefit from aspirin use; however, current studies are inconclusive due to important limitations, such as the narrow geographical scope of participants, which limits genetic diversity and may affect the applicability of the results to broader populations [31].

On the other hand, patients who underwent percutaneous coronary intervention (PCI) with drug-eluting stents (DESs) and had Lp(a) levels ≥ 30 mg/dL were evaluated based on the hypothesis that prolonged dual antiplatelet therapy (DAPT) beyond the standard one-year recommendation might confer an additional benefit in this subgroup. Over a median follow-up of 2.4 years post-procedure, the study by Cui et al. demonstrated a significant reduction in both major adverse cardiovascular events (MACEs) and major adverse cerebrovascular and cardiovascular events (MACCEs), without a corresponding increase in clinically significant bleeding risk [32].

## 6. Lp(a) Assessment

The advantage of the traditional lipid profile, besides its proven efficacy, lies in its cost-effectiveness and the relative simplicity of the available methods, compared with Lp(a) determination, which is more expensive and less standardized [33]. However, recent studies have also linked atherosclerosis to inflammation and immune processes, suggesting that a high LDL cholesterol level is no longer the only driver for developing plaques, and the accumulation of lipids in the arterial wall is not at all a passive phenomenon [23,34,35,36]. A study conducted by the National Institutes of Health in collaboration with the International Federation of Clinical Chemistry evaluated a proposed reference material to standardize Lp(a) measurements across various methods. While calibration using the reference material improved uniformity in control samples, significant variability in measurements persisted due to the heterogeneity in apo(a) size, undermining the accuracy of risk assessments for coronary artery disease [37]. Even though we might be able to face this challenge, regarding the variability in apo(a) size, we still have other obstacles ahead, like the difference between mass-based (mg/dL) and molar-based (nmol/L) assays. Conversion between these units remains imprecise, with commercial kits still widely using less reliable mass-based measurements [38].

Both immunoturbidimetry and nephelometry use turbidity measurements to determine Lp(a), with slight differences. While turbidimetry measures turbidity by analyzing light transmission, nephelometry relies on light scattering. Specific reagents can be used to improve the accuracy of these methods. Although they provide broad measurement ranges, they are less sensitive to the variability in apo(a) size [39].

Radial immunodiffusion (RID), or the Mancini method, is a straightforward immunoassay that measures Lp(a) by observing the precipitate formed during the antigen–antibody reaction. Although it can detect Lp(a) concentrations as low as 1.5 mg/dL, it cannot assess the size variability of apo(a). A double-antibody radioimmunoassay, which improves sensitivity, has been developed but is more time-consuming, complex and prone to higher variability [40].

Electrophoresis techniques, including immunofixation electrophoresis, counterimmunoelectrophoresis and rocket immunoelectrophoresis, are commonly used for the analysis of Lp(a) concentrations in serum, but they have the same limitations as those previously listed. While these methods offer sensitivity and allow for quantification, they are unable to measure the size variability of Lp(a) or its heterogeneity. Liquid chromatography–mass spectrometry (LC-MS/MS) has emerged as a promising tool for Lp(a) quantification, providing accurate results and better standardization compared to the traditional methods [41].

Commercial immunoassays are widely used for the high-throughput measurement of Lp(a), but their accuracy is compromised by sensitivity to apo(a) isoform size variations, which can lead to inconsistent results. Monoclonal antibody-based ELISA assays are more reliable for assessing the relationship between Lp(a) and cardiovascular disease risk, as they are less affected by isoform size variability, providing more consistent and accurate measurements. Denka-based assays, developed by Denka Seiken Co., Ltd. (Tokyo, Japan), are considered the least affected by isoforms among commercially available options due to their use of multiple calibrators that cover a broad range of apo(a) isoform distributions, thus ensuring more accurate Lp(a) measurements [40].

Szarek et al. [42] compared three methods for measuring lipoprotein(a): two immunoassay-based methods (IA-mass and IA-molar) and one mass spectrometry (MS)-based method. Samples were collected, frozen and analyzed using standardized procedures. Immunoassay methods used antibodies and calibration standards, while the MS method was isoform-independent and directly quantified specific peptides from apolipoprotein(a). The study found that all three methods were similarly effective at predicting cardiovascular risk, specifically the likelihood of MACEs, in patients with recent acute coronary syndrome receiving high-intensity or maximum-tolerated statins. The MS-based method correlated better with the molar immunoassay than with the mass-based immunoassay, but differences between the molar methods highlighted a lack of full equivalence (Table 1). Despite this, all three tests showed comparable utility for assessing cardiovascular risk and evaluating treatment benefits with alirocumab [42].

In a multicenter cross-sectional epidemiological study conducted by Nissen S et al. [7], 48,135 patients across 48 countries with documented ASCVD were analyzed. Given that only almost one in ten (13.9%) of the subjects had prior Lp(a) measurements, it can be inferred that screening for Lp(a) in individuals with established atherosclerosis is insufficient. This suggests that screening in otherwise healthy individuals may be even less frequent, although further studies would be needed to confirm this. Furthermore, the study revealed that over 25% of those who had their Lp(a) levels measured exhibited values above 50 mg/dL (or 125 nmol/L), a threshold that may be linked to the etiology of their ASCVD [7].

A survey carried out by the European Atherosclerosis Society (EAS) Lipid Clinic network had the aim of finding out when and how lipoprotein(a) is measured in clinical practice. Almost one quarter (24.5%) of the practitioners included in the survey stated that they do not routinely check Lp(a), the reasons being that the cost is too high and the fact that there is a lack of treatment options [8]. This observation should prompt action to improve the availability of Lp(a) measurement and to further research the potential use of PCSK9 inhibitors for their proven Lp(a)-lowering effect, at least until the use of nucleic acid-based gene-silencing therapies that specifically target apo(a) gene transcripts become readily available [6,43,44,45,46].

In the 2019 ESC guidelines for managing dyslipidemias, there is a class IIa level C recommendation for screening Lp(a) at least one time in every individual’s life, comparing the risk of very elevated Lp(a) with familial hypercholesterolemia. This recommendation is especially directed towards those with early-onset CVD, recurrent CVD despite optimal medical treatment, a family history of premature CVD or elevated Lp(a), aortic stenosis or a very high cardiovascular risk according to Framingham or ESC Systematic Coronary Risk Evaluation (SCORE). If the recommendations for the target population are reasonably standardized, the guidelines for follow-up are less so. There should be a more aggressive control of traditional risk factors (e.g., LDL, smoking, diabetes). However, no specific recommendations address elevated Lp(a) levels [38,47].

## 7. Treatment Options

### 7.1. Niacin

Some treatment options like niacin have, indeed, inconclusive evidence. In a meta-analysis conducted by Sahebkar A. et al. [48], the effect of extended-release nicotinic acid (ER niacin) on plasma Lp(a) concentrations was assessed using data from 14 randomized placebo-controlled trials, including 9013 subjects. The treatment was associated with a significant reduction in Lp(a) levels, with a weighted mean difference of −22.90% (95% CI: −27.32, −18.48, *p* < 0.001). The reduction was consistent across different dose ranges (<2000 mg/day and ≥2000 mg/day) [48]. Schwartz et al. published a review in 2022 that concluded that the lowering effect of niacin on Lp(a) has no or a slight benefit at best [49].

### 7.2. Statins

Statins, as potent inhibitors of HMG-CoA reductase, effectively reduce LDL-c levels and cardiovascular risk in both primary and secondary prevention; however, their impact on Lp(a) appears minimal, with studies indicating a slight increase in Lp(a) levels, particularly with high-intensity statin therapy [50]. A systematic review and meta-analysis of 39 randomized trials, published in 2022 (24,448 participants), assessed the impact of statins on Lp(a) levels compared to placebo. The pooled data indicated minimal absolute changes (1.1 mg/dL, 95% CI: 0.5–1.6, *p* < 0.0001) and no significant percentage changes (0.1%, 95% CI: −3.6% to 4.0%, *p* = 0.95) [51].

### 7.3. Lp(a) Apheresis

Víšek et al. followed 14 familial hypercholesterolemia (FH) patients for 15 years, with the aim of evaluating the efficacy of LA in preventing cardiovascular disease progression. The results were promising, with LA significantly reducing cholesterol levels, inflammation markers and endothelial dysfunction in patients with familial hypercholesterolemia (FH), both immediately after procedures and over time. One of the studies’ key limitations is that all patients were also treated with high-dose statins, ezetimibe, and PCSK9 inhibitors, which could have contributed to the observed benefits, making it difficult to isolate the effects of LA alone [52,53]. Schettler et al. [54] demonstrated that lipoprotein apheresis (LA) reduces both major adverse cardiovascular events (MACEs) and major adverse non-coronary events (MANCEs) in patients with baseline Lp(a) levels > 60 mg/dL and LDL-C < 100 mg/dL. However, despite the significant risk reduction observed, it remains uncertain whether the benefits can be solely attributed to Lp(a) lowering, given that the LDL-C levels did not meet the currently recommended target of <55 mg/dL for high-risk patients [54]. The recommendation between different guidelines varies, and the reason why it is not that frequently used in practice, in spite of the proven benefits, is the unavailability and the complexity of the method [55,56,57].

### 7.4. PCSK9-i

The Odyssey Outcomes trial was a multicenter, double-blind, randomized trial that evaluated 18,924 patients with recent acute coronary syndrome and elevated LDL cholesterol levels despite high-intensity statin therapy. Participants received either alirocumab or placebo every two weeks, targeting an LDL cholesterol level of 25–50 mg/dL, with the primary endpoint being a composite of major adverse cardiovascular events such as death from coronary heart disease, myocardial infarction, ischemic stroke, or unstable angina requiring hospitalization. The study highlighted that alirocumab, a PCSK9 inhibitor, significantly reduced both mortality and cardiovascular events, particularly in patients with inadequately controlled LDL cholesterol levels. Alirocumab also demonstrated a 20–25% reduction in Lp(a) levels (samples were analyzed with an immunoturbidimetric assay), potentially contributing to these effects. Additional research is essential to fully understand the pathways responsible for these extended cardiovascular benefits. The inclusion criteria are a major limitation of the study—only patients with a history of acute coronary syndromes were included, meaning that the results might not apply to the general population [5,47].

### 7.5. Nucleic Acid-Based Gene Silencing

Nucleic acid-based gene silencing, involving antisense oligonucleotide (ASO) or short-interfering ribonucleic acid (siRNA) strategies, represents a new and more advanced approach for suppressing the intracellular production of PCSK9 and apo(a) [46,58]. Antisense oligonucleotides are usually administered subcutaneously and act on the cell nucleus by inhibiting the transcription of a specific protein, such as PCSK9 and apo(a), which further decreases the amount of LDL cholesterol or Lp (a) produced by the liver. One example of such a drug is pelacarsen, which targets the production of apo(a) [46,59]. SiRNA therapy uses conjugated nucleic acids that act by suppressing a specific gene’s expression, interfering with the translation and synthesis of new proteins. Inclisiran is an siRNA drug that targets PCSK9, while olpasiran is also part of the same class but targets the apo(a) of the *LPA gene* [46,60,61].

#### 7.5.1. Pelacarsen

A phase 2B trial evaluated the impact of pelacarsen, a hepatocyte-targeted antisense oligonucleotide, on lowering Lp(a) in 286 patients with cardiovascular disease and elevated baseline Lp(a) levels (≥60 mg/dL). Participants received pelacarsen in varying regimens or a placebo for 6–12 months, with Lp(a) levels assessed at baseline, 6 months and 16 weeks post-treatment, using an isoform-independent assay, which involves plasma being added to monoclonal antibody LPA4-conjugated magnetic beads in the presence of proline and epsilon-aminocaproic acid. Pelacarsen demonstrated a dose-dependent efficacy, with mean Lp(a)-C reductions ranging from 29% to 67% compared to a minimal 2% decrease in the placebo group. An advanced assay allowed precise Lp(a)-C measurements with a high sensitivity and broad applicability across population Lp(a) levels. Temporal trends revealed consistent reductions in Lp(a), though levels partially rebounded after discontinuation of therapy [62]. The findings suggest that pelacarsen offers a promising approach to managing elevated Lp(a)-mediated cardiovascular risks, with further research needed to explore long-term outcomes. A phase 3, double-blind, randomized, placebo-controlled, multicenter trial known as Lp(a) HORIZON is currently underway to evaluate the safety and effectiveness of pelacarsen [63].

#### 7.5.2. Inclisiran

In ORION studies, inclisiran consistently lowered Lp(a) by approximately 15–26%, with greater reductions in two-dose regimens and long-term treatment. These reductions were more pronounced compared to the placebo, though the effect on Lp(a) was less pronounced than its effects on LDL-C and apoB levels. These findings align with those from studies evaluating PCSK9 inhibitors, such as the ODYSSEY OUTCOMES trial (alirocumab) and the FOURIER study (evolocumab). Although inclisiran’s mechanism of action differs, these drugs ultimately target the same enzyme. This highlights inclisiran’s potential in addressing Lp(a)-mediated cardiovascular risks, especially when used alongside other lipid-lowering therapies [5,64,65].

#### 7.5.3. Olpasiran

In a randomized, placebo-controlled trial involving patients with atherosclerotic cardiovascular disease and an elevated Lp(a) (>150 nmol/L), olpasiran significantly reduced Lp(a) concentrations across all doses. At 36 weeks, placebo-adjusted reductions ranged from −68.5% with the 10 mg dose every 12 weeks to −100% with the 225 mg dose every 12 weeks. Additionally, 67% to 100% of patients achieved Lp(a) levels of <125 nmol/L depending on the dose and regimen. The trial included only patients with documented ASCVD and a serum level of Lp(a) of at least 150 nmol/L, or approximately 70 mg/dL, so the results might not apply to the general population or subjects with a slight increase in Lp(a). Also, there is no mention of what specific method was used for determining the concentration of Lp(a), which is an important factor given the various isoforms of apo(a) that can affect the measurement [45].

#### 7.5.4. Lepodisiran

Phase 1 clinical trials, using the Roche immunoassay, demonstrated the efficacy and safety of lepodisiran, enabling its progression to phase 2 trials [66]. Lepodisiran showed excellent tolerability and produced significant, dose-dependent reductions in serum lipoprotein(a) levels (a reduction in mean value from 41% in the 4 mg group, to 97% in the 608 mg group), with effects sustained over a prolonged duration (a 94% median reduction in lipoprotein(a) levels was observed at day 337 in the group receiving the highest dose.). These promising results highlight the potential of lepodisiran and support its further clinical evaluation, to see whether the decrease in Lp(a) translates into lowering the CVD risk [67].

#### 7.5.5. Small-Molecule Inhibitors

**Figure 1:**
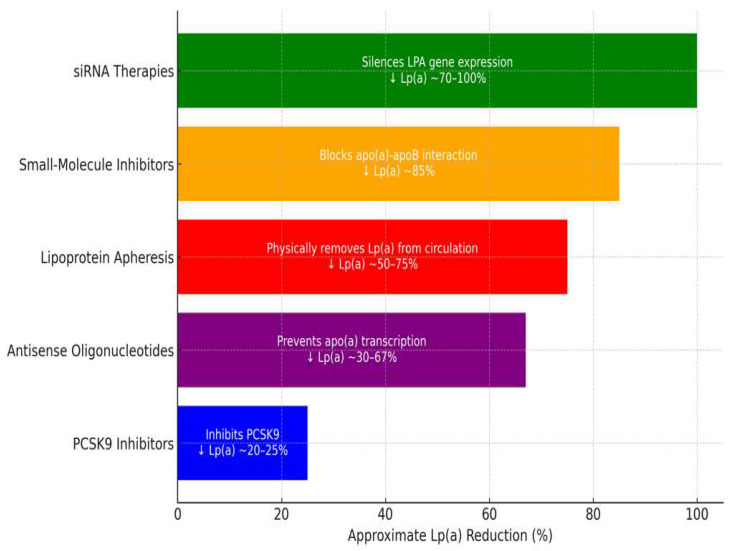
Therapies targeting Lp(a) reduction (adapted from reviewed literature).

While adverse effects associated with Lp(a)-lowering therapies are rare and typically limited to injection-site reactions, the need for more convenient and cost-effective treatments has driven the development of oral alternatives (Table 2 and Figure 1). One such therapy is muvalaplin, a novel oral small-molecule inhibitor. Muvalaplin disrupts the interaction between apolipoprotein B-100 and apolipoprotein(a), a critical step in the assembly of Lp(a), thereby significantly reducing circulating Lp(a) concentrations [68]. In the KRAKEN trial, muvalaplin demonstrated a remarkable efficacy, with mean Lp(a) levels reduced by up to 85.8% in participants receiving the highest therapeutic dose (240 mg daily). These reductions are considered clinically significant and have positioned muvalaplin as a promising candidate for addressing an elevated Lp(a). However, several limitations of this trial warrant attention. First, the study employed commercial assays for Lp(a) measurement, which may underestimate the true magnitude of Lp(a) lowering. Additionally, the trial’s short duration (12 weeks) limits insights into long-term safety and efficacy. Importantly, it remains to be determined whether the observed Lp(a) reductions will translate into meaningful reductions in cardiovascular (CV) risk. Muvalaplin is currently undergoing phase 3 clinical trials to further evaluate its safety, efficacy and potential role in reducing cardiovascular events. If successful, it could provide a significant advancement in the management of elevated Lp(a), particularly for patients seeking non-injectable treatment options [69].

## 8. Discussions

**Figure 2:**
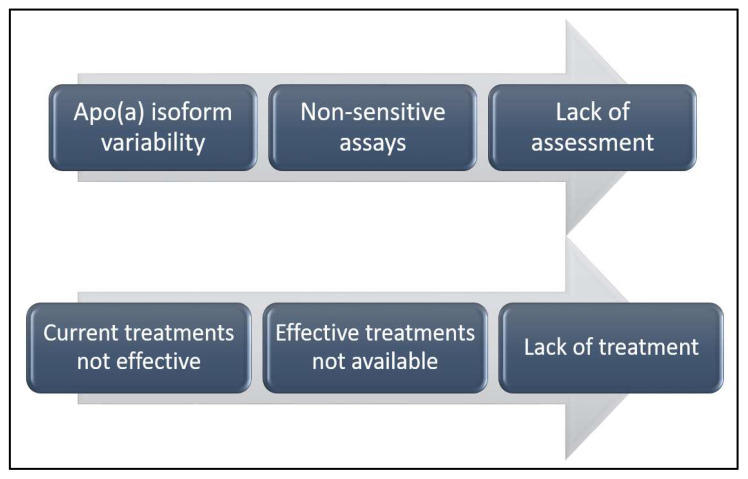
Current problems regarding Lp(a) (adapted from reviewed literature).

**Figure 3:**
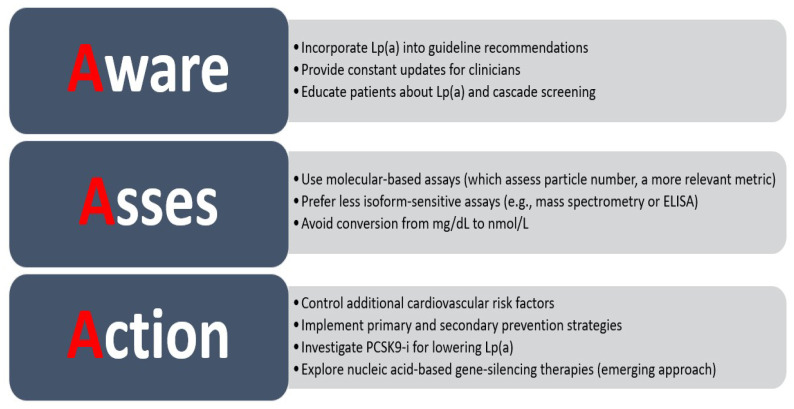
Triple A—solutions to overcome challenges in Lp(a) screening and treatment (adapted from reviewed literature).

Although identifying individuals with elevated Lp(a) levels may initially pose a challenge, cascade screening has been proposed as a practical and effective approach once an index case is identified (Figure 2 and Figure 3). Given the genetic basis of Lp(a) elevation, first- and second-degree relatives are likely to exhibit similarly high levels, even in children. In a study published in 2021 by Chakraborty et al. [70], elevated Lp(a) levels (≥50 mg/dL and ≥100 mg/dL; approximately 125 nmol/L and 250 nmol/L, respectively) were identified in one new case for every 1.5 and 2.8 relatives screened. When using higher proband case thresholds of 100–150 mg/dL and ≥150 mg/dL (∼250–375 nmol/L and ≥375 nmol/L), elevated Lp(a) levels of ≥50 mg/dL (∼125 nmol/L) were found in 1 out of every 1.8 and 1.2 relatives tested, respectively. Implementing cascade screening could facilitate the early identification of at-risk individuals, allowing for a more aggressive management of other cardiovascular risk factors and potentially delaying the onset of atherosclerotic cardiovascular disease [70,71].

While therapies such as niacin and statins have not demonstrated efficacy in lowering Lp(a), and others, like siRNA, ASO and muvalaplin, are not yet widely available, PCSK9 inhibitors are accessible treatments with proven Lp(a)-lowering effects. Further studies are required to assess whether this effect could warrant a new indication for this class of medications, independent of LDL cholesterol levels, particularly in high-risk areas, as in those in Eastern Europe, allowing patients to bypass the process of trying statins, ezetimibe and other treatments, which could otherwise delay the benefits [72].

## 9. Conclusions

In summary, although Lp(a) is gaining recognition as a significant marker for ASCVD, its routine use in clinical practice remains limited due to challenges related to measurement accuracy and the limited availability of effective treatments. Future research should focus on improving Lp(a) quantification techniques and evaluating the long-term efficacy and safety of gene-silencing therapies. Additionally, further studies are needed to explore the potential of PCSK9 inhibitors to reduce cardiovascular risk in patients independently of LDL levels. With the development of these novel therapies, Lp(a) may soon become a critical target in managing atherosclerotic cardiovascular disease.

## Figures and Tables

**Table 1:** Lp(a) assessment methods (adapted from reviewed literature).

Assesment Method	Description	Advantages	Limitations	Cut-Off Value
Immunoturbidimetric Assay	Measures turbidity changes due to Lp(a)–antibody complexes	Broad range; High-throughput	Influenced by apo(a) size; Variability between reagents	LOW
Nephelometry	Measures light scattering from antigen–antibody complexes	Automated; Reproducible; Broad detection range	Influenced by apo(a) size; Less standardization	<30 mg/dL or <75 nmol/L
ELISA	Uses antibodies targeting Lp(a) components	Sensitive; Adaptable	Accuracy depends on antibody used	INCREASED RISK
Denka Assay	Commercial assay with multiple calibrators for isoform coverage	Least affected by isoform heterogeneity; More accurate and standardized	Dependent on proper calibration; Available through specific providers	30–50 mg/dL or 75–125 nmol/L
Radial Immunodiffusion	Simple diffusion-based immunoassay measuring precipitate ring	Low cost; Detects low levels of Lp(a)	Low sensitivity; Slow; Cannot assess apo(a) isoform size	HIGH >50 mg/dL or >125 nmol/L
Mass Spectrometry	Direct quantification of apolipoprotein(a) peptides	Isoform-independent; Accurate; Better standardization	Expensive; Not widely available; Complex technique	

**Table 2:** Therapies targeting Lp(a) reduction (adapted from reviewed literature).

Therapy	Mechanism of Action	Reduction in Lp(a)	Duration of Effect	Limitations
PCSK9-i	Increases clearance via LDLR upregulation	20–25%	Short-term, frequent dosing	Moderate effect on Lp(a) reduction
ASOs (Pelacarsen)	Inhibits apo(a) mRNA translation	29–67%	Long-term, monthly dosing	Not yet widely available
siRNA (Olpasiran)	Blocks apo(a) mRNA translation	68.5–100%	Long-term, quarterly dosing	Not yet widely available
Apheresis	Physically removes Lp(a) from circulation	up to 73%	Immediate but transient	Invasive, expensive
Small-molecule inhibitors (Muvalaplin)	Disrupts Lp(a) assembly	up to 85.8%	Oral, daily dosing	Early-stage development

## Data Availability

No new data were created or analyzed in this study. Data sharing is not applicable.
